# Perillaldehyde Inhibition of cGAS Reduces dsDNA-Induced Interferon Response

**DOI:** 10.3389/fimmu.2021.655637

**Published:** 2021-04-22

**Authors:** Lei Chu, Chenhui Li, Yongxing Li, Qiuya Yu, Huansha Yu, Chunhui Li, Wei Meng, Juanjuan Zhu, Quanyi Wang, Chen Wang, Shufang Cui

**Affiliations:** ^1^ State Key Laboratory of Natural Medicines, School of Life Science and Technology, China Pharmaceutical University, Nanjing, China; ^2^ Experimental Animal Center, Shanghai Pulmonary Hospital, Tongji University School of Medicine, Shanghai, China

**Keywords:** cyclic GMP-AMP synthase (cGAS), perillaldehyde, herpes simplex virus type 1 (HSV-1), innate immunity, autoimmune disease

## Abstract

Cyclic GMP-AMP synthase (cGAS), serving as a primary sensor of intracellular DNA, is essential to initiate anti-microbial innate immunity. Inappropriate activation of cGAS by self-DNA promotes severe autoinflammatory diseases such as Aicardi–Goutières syndrome (AGS); thus, inhibition of cGAS may provide therapeutic benefit in anti-autoimmunity. Here we report that perillaldehyde (PAH), a natural monoterpenoid compound derived from *Perilla frutescens*, suppresses cytosolic-DNA-induced innate immune responses by inhibiting cGAS activity. Mice treated with PAH are more susceptible to herpes simplex virus type 1 (HSV-1) infection. Moreover, administration with PAH markedly ameliorates self-DNA-induced autoinflammatory responses in a mouse model of AGS. Collectively, our study reveals that PAH can effectively inhibit cGAS-STING signaling and could be developed toward the treatment of cGAS-mediated autoimmune diseases.

## Introduction

The innate immune system is initiated as the front-line defense against microbes or damaged self-component *via* germline-encoded pattern-recognition receptors (PRRs) (1). cGAS is a key intracellular DNA sensor that catalyzes the conversion of GTP and ATP to cyclic GMP-AMP (2′3′-cGAMP), which serves as a second messenger to bind and activate the endoplasmic adaptor protein stimulator of interferon genes (STING) (2, 3). cGAMP binding induces a conformation change and trafficking of STING, which activates the downstream effectors TANK-binding kinase 1 (TBK1) and interferon regulatory factor 3 (IRF3). Subsequently, phosphorylated IRF3 dimerizes and translocates into the nucleus to produce type I interferon (IFN) and other proinflammatory cytokines (4–6).

While detection of foreign DNA plays an indispensable role in pathogen defense, aberrant activation of cGAS by self-DNA can promote severe autoimmune diseases. Trex1 is a DNA 3′ repair exonuclease that degrades cytosolic DNA, and deficiency of Trex1 leads to the accumulation of cytosolic DNA (7, 8). Loss-of-function mutations in *Trex1* have been identified in autoimmune disorders such as Aicardi–Goutières syndrome (AGS) and familial chilblain lupus in human patients (9, 10). *Trex1^−/−^* mice develop the severe early onset systemic autoinflammatory disease with a short lifespan of 2–3 months (8). Deletion of *Cgas* is sufficient to suppress the autoimmune disease phenotype in the *Trex1^−/−^* mouse model of AGS (11). In addition, increased cGAS mRNA levels and production of cGAMP have been observed in Systemic Lupus Erythematosus (SLE) patients, indicating that cGAS-STING signaling is also involved in a subset of SLE patients. These observations together demonstrate the importance of cGAS inhibition in treating cGAS-mediated autoimmunity and that cGAS may be a potential drug target for preventing autoinflammation.

Natural products have long been valuable resources for discovering and developing novel molecules to treat various human diseases (12). *Perilla frutescens* has been used as an ingredient in Chinese herbal medicine. By suppressing proinflammatory cytokines and inducing anti-inflammatory cytokines, *Perilla frutescens* leaf extract effectively ameliorates dextran sulfate sodium (DSS)-induced colitis (13). Volatile monoterpenoid Perillaldehyde (PAH), the major component and the most effective ingredient of *Perilla frutescens* leaf (14), is biologically active and exhibits anti-inflammatory (13, 15, 16), antifungal activity (17, 18), antioxidant (19), and anti-tumor effects (20, 21). Despite the multi-functionality of PAH, the effect of PAH in innate immunity has not been characterized well. In this study, we report that PAH specifically impairs the cytosolic DNA-induced innate signaling and inflammatory responses by inhibiting the activity of cGAS. PAH suppresses type I IFN and IFN stimulated gene (ISGs) expression triggered by HT-DNA but not poly(I:C) or cGAMP. Mice treated with PAH are more susceptible to herpes simplex virus type 1 (HSV-1) infection. Furthermore, PAH administration alleviates autoinflammatory responses in *Trex1^−/−^* bone marrow-derived macrophages (BMDMs) and the autoimmune disorder in *Trex1^−/−^* mice.

## Materials and Methods

### Mice

*Trex1^+/−^* mice were kindly licensed by Dr. Tomas Lindahl and Dr. Deborah Barnes (Cancer Research UK, London) and provided by Dr. Nan Yan (University of Texas Southwestern Medical Center). *Trex1^−/−^* mice were generated by further mating the male and female *Trex1^+/−^* mice and were genotyped by standard PCR. All mice used in this study were on C57BL/6 background. 4 weeks old *Trex1^−/−^* mice were started to drug administration. All mice were maintained under specific pathogen-free (SPF) circumstances at the Center for New Drug Safety Evaluation and Research, China Pharmaceutical University. All animal works were carried out following the National Institutes of Health Guide for the Care and Use of Laboratory Animals.

### Cell Culture and Transfection

MEF, HFF, and Vero cells were cultured in DMEM medium (Invitrogen), supplemented with 10% FBS (Gibco) and 1% penicillin–streptomycin (Invitrogen). L929 cells and BMDMs were cultured in RMPI-1640 (Gibco) plus 10% FBS and 1% penicillin–streptomycin. BMDMs were generated as previously reported (22). All cells were cultured at 37°C and 5% CO_2_. Lipofectamine 3000 (Invitrogen) was used for transfection according to the manufacture’s procedure. cGAMP stimulation was performed with a permeabilization buffer (50 mM HEPES pH 7, 100 mM KCl, 3 mM MgCl_2_, 0.1 mM DTT, 85 mM sucrose, 0.2% BSA, 1 mM ATP, and 0.1 mM GTP with 10 μg•ml^−1^ digitonin (Sigma-Aldrich)) at 37°C for 30 min, followed by replacing with fresh medium.

### Virus

HSV-1, GFP-HSV-1, VSV, and GFP-VSV were propagated and titrated by standard plaque assay on Vero cells.

### Reagents and Antibodies

PAH, CMC-Na, Poly(I:C), and HT-DNA were purchased from Sigma-Aldrich. cGAMP was obtained from Invivogen. ISD was prepared by annealing equimolar amounts of sense and antisense DNA oligonucleotides at 95°C for 10 min, then cooled to room temperature. Antibodies: anti-IRF3 (CST, #4302), anti-Phospho-IRF3 (CST, #4947), anti-TBK1 (Abcam, ab40676), anti-Phospho-TBK1 (CST, #5483), anti-cGAS (CST, #31659), anti-STING (CST, #13647), anti-RIG-I (CST, #4200), anti-MAVS (CST, #4983), anti-Histone H3 (CST, #4499), anti-GAPDH (Santa Cruz, sc-32233), anti-ISG15 (Santa Cruz, sc-166755), anti-Rabbit IgG (H+L) (Jackson, 111-035-003), anti-Mouse IgG (H+L) (Jackson, 115-035-003).

### Western Blot Analysis

Cell pellets were collected and lysed with lysis buffer (50 mM Tris-HCl pH 7.5, 150 mM NaCl, 1% Triton X-100) containing 1× EDTA-Free Protease inhibitor cocktail (Roche) and 1 mM PMSF (Sigma-Aldrich). The resuspended cell pellets were incubated on ice for 30 min, followed by centrifugation at 12,000 rpm for 15 min at 4°C. The supernatants were collected for SDS-PAGE. The bands were probed with indicated primary and secondary antibodies and visualized using a SuperSignal West Pico chemiluminescence ECL kit (Pierce).

The native PAGE assay was performed as described previously (23). Briefly, cell pellets were lysed with native lysis buffer (50 mM Tris-HCl pH 7.5, 150 mM NaCl, 1% NP-40, 1× EDTA-Free Protease inhibitor cocktail, 1 mM PMSF, and 1mM orthovanadate). The resuspended cell pellets were incubated on ice for 30 min followed by centrifugation at 12,000 rpm for 15 min at 4°C. The supernatants were collected for native sample preparation. After pre-run in the electrophoresis buffer (25 mM Tris-HCl, pH 8.4, 192 mM glycine with and without 0.2% deoxycholate in the cathode and anode chamber, respectively), native samples were loaded to gel and electrophorese for 60 min at 25 mA. Then the gel was soaked into 0.1% SDS-containing electrophoresis buffer for 30 min at room temperature, followed by standard immunoblot analysis.

For subcellular fraction assay, cell pellets were lysed with buffer A (10 mM HEPES pH 7.9, 1.5 mM MgCl_2_, 1 mM KCl, 0.5 mM DTT, and 1 mM PMSF), then 0.2% NP-40 was added after incubation on ice for 15 min. The supernatants were collected as the cytoplasmic fraction after centrifugation for 5 min at 4,000 rpm. The pellets were washed twice with buffer A and then resuspended with buffer C (20 mM HEPES, pH 7.9, 25% glycerol, 0.42 M sodium chloride, 1.5 mM MgCl_2_, 0.2 mM EDTA, 0.5 mM DTT, and 2 mM PMSF). After incubation on ice for 0.5–2 h, the supernatants were collected as the nucleic fraction by centrifugation for 15 min at 12,000 rpm. The cytoplasmic and nucleic fractions were analyzed by standard immunoblot analysis.

### Immunofluorescence and Confocal Microscopy

Cells were fixed for 15 min with 4% paraformaldehyde in PBS, washed with PBS, and permeabilized with 0.25% Triton X-100 in PBS for 20 min, then washed and blocked with 5% BSA for 1 h. After incubation with indicated primary antibodies overnight at 4°C, fluorescent-conjugated secondary antibodies were incubated for 2 h at room temperature. Slides were mounted with a fluorescent mounting medium (Dako) after DAPI (Sigma-Aldrich) staining and captured using a confocal microscope (LSM800, Carl Zeiss) with a 63× oil objective.

### RNA Isolation and Quantitative PCR Analysis

Total RNA from cells was isolated with TRIzol reagent (Invitrogen) according to the manufacturer’s instruction. cDNA was synthesized with 0.5 μg RNA and HiScript III Q RT SuperMix for qPCR (Vazyme). Real-time PCR was performed using ChamQ SYBR qPCR Master Mix (Low ROX Premixed) (Vazyme). Data were normalized to the expression of mouse *Gapdh* or human *GAPDH* through the comparative Ct method (2-^△△Ct^).

### MTS Assay

According to the manufacturer’s instructions, cell viability was measured by CellTiter 96 AQueous One Solution Cell Proliferation Assay (MTS) kit (Promega).

### ALT and AST Analysis

The levels of aspartate aminotransferase (AST) and alanine aminotransferase (ALT) in serum were measured by Siemens Dimension^®^ Xpand Plus™ biochemical autoanalyzer.

### Detection of Autoantibodies

The hearts from WT mice were lysated in RIPA lysis buffer. The protein extractions were separated by SDS-PAGE and transferred to PVDF membrane, and then incubated with 1:200 dilution of serum overnight at 4°C. Autoantibodies were detected with horseradish peroxidase conjugated anti-mouse IgG.

### Enzyme-Linked Immunosorbent Assay

Concentrations of the cytokines in culture supernatants or serum were measured by ELISA kit (4A Biotech, CME0116) according to the manufacturer’s instructions.

### Hematoxylin and Eosin

Tissues were fixed in 4% paraformaldehyde, paraffin-embedded, cut into 5 mm sections, and stained with hematoxylin and eosin.

### Viral Infection

Viral infection assays were executed as described in the figures. Briefly, 8–12 weeks old WT C57BL/6 mice (16–22 g, initially) were orally administered with vehicle (0.03% CMC-Na solution) or PAH at 120 mg•kg^−1^ body weight once per day for up to 2 weeks. The body weight of each mouse was measured daily. The mice were injected intravenously with HSV-1 for 12 h after PAH administration. Then the serum and tissues were collected for analysis of RNA and cytokines. For a durable anti-infection study, HSV-1-infected mice were monitored for another 7 days with drug administration. Meanwhile, the body weight of each mouse was measured daily.

### Flow Cytometry

L929 cells seeded in 12-well plates were treated with vehicle or PAH (200 μM) for 6 h, and then infected with GFP-HSV-1 (MOI = 0.3) or GFP-VSV (MOI = 0.4) for 16 h. Cell populations were suspended in PBS buffer after trypsin digestion. Then, the ratios of GFP-positive cells were analyzed by flow cytometry (Attune NxT Flow Cytometer; Thermo Fisher Scientific). In flow cytometry gating, cells were first gated for single cells (forward scatter area *versus* side scatter area) and further analyzed for their GFP expression. The results were analyzed by FlowJo.

### Plaque Assay

The titers of the virus in the supernatant were determined by standard plaque assay with Vero cells. After infection with virus-contained supernatant for 2 h, cell monolayers were washed twice with pre-warmed DMEM medium, followed by incubation with a mixed media (1× MEM, 2% FBS, 1% Low Melting Point Agarose) until the formation of the plaques. Then the cells were fixed with 4% paraformaldehyde and stained with crystal violet. The titers of the virus were measured by counting the plaques.

### cGAS Enzymatic Activity Assay *In Vitro*

cGAS activity was analyzed as described previously (24). The enzymatic activity of cGAS was measured with purified recombinant full-length cGAS in 100 μl reaction buffer (2 mM cGAS, 0.2 mM ATP, 0.2 mM GTP, 100 mM NaCl, 40 mM Tris-HCl pH 7.5, 1 mM MgCl_2_ and 0.01 mg•ml^−1^ dsDNA). Then the reaction buffer was heated at 99°C to denature proteins. After centrifugation at 12,000 g for 10 min, the supernatants were collected and extracted with ACQUITYUPLC^®^ BEH Amide Colum, which was previously equilibrated with running buffer (1% Formic acid solution). All samples were analyzed using a Waters XEVO^®^ TQD system (Waters Corp.) equipped with electrospray ionization (ESI).

### Statistical Analyses

Statistical analyses were performed with GraphPad Prism 8. A standard two-tailed unpaired Student’s t-test was performed for statistical analyses of two groups or as indicated in the figure legends. All analyzed data are expressed as mean ± standard deviation (SD). Differences with a *P*-value <0.05 were considered significant.

## Results

### PAH Inhibits Cytosolic DNA-Triggered Type I IFN Production

**Figure 1:**
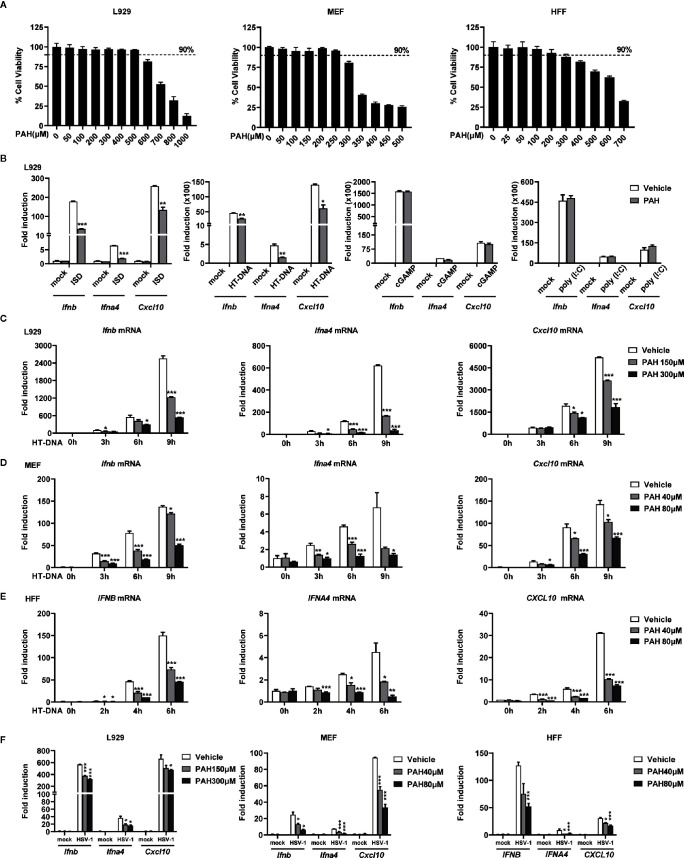
PAH inhibits cytosolic DNA-triggered type I IFNs production. **(A)** L929, MEF, and HFF cells were treated with indicated PAH concentrations for 24 h, and then the cell proliferation was measured by MTS assay. **(B)** L929 cells were treated with Vehicle or PAH (300 μM) for 6 h and then stimulated with ISD (4 μg•ml^−1^), HT-DNA (4 μg•ml^−1^), cGAMP (1 μg•ml^−1^), or poly(I:C) (3 μg•ml^−1^). The induction of *Ifnb*, *Ifna4*, and *Cxcl10* mRNA expression was then measured by qPCR. **(C–E)** L929 **(C)**, MEF **(D)**, or HFF **(E)** cells were treated with Vehicle or PAH for 6 h and then stimulated with HT-DNA (4 μg•ml^−1^) for the indicated times. The induction of *Ifnb*, *Ifna4*, and *Cxcl10* mRNA expression was then measured by qPCR. **(F)** L929, MEF, or HFF cells were treated with Vehicle or PAH for 6 h and then infected with HSV-1 (MOI = 1) for 6 h (L929 and MEF) or 3 h (HFF). The induction of *Ifnb*, *Ifna4*, and *Cxcl10* mRNA expression was then measured by qPCR. All of the experiments were repeated at least three times. Data in **(A–F)** are presented as mean ± SD. *P < 0.05, **P < 0.01, ***P < 0.001.

To exclude the potential cytotoxicity effect of PAH on the innate immune system, we first determined the *in vitro* cytotoxicity of PAH on different cells. Two murine cell lines, L929 (murine fibrosarcoma cells) and MEF (mouse embryonic fibroblasts) and one human cell line HFF (human foreskin fibroblasts), were incubated with different doses of PAH for 24 h, and then the cell viability was quantified by MTS assay. There was no significant cytotoxic side effect for L929, MEF, and HFF at the dose of 500, 250, and 200 μM, respectively (**Figure 1A**). Next, we incubated L929 cells with 300 μM PAH and measured the induction of type I IFNs and downstream genes upon various stimuli. PAH markedly inhibited the IRF3-responsive genes (*Ifnb*, *Ifna4*, and *Cxcl10*) expression stimulated by DNA mimics ISD or HT-DNA (herring testis DNA), whereas did not affect these antiviral genes expression triggered by poly(I:C) (a ligand of RNA sensor) or cGAMP (2′3′-cGAMP, a ligand that directly activates STING) (**Figure 1B**).

To substantiate the specific inhibitory effect of PAH on cytosolic DNA-induced type I IFN expression, we incubated L929, MEF, and HFF cells with different doses of PAH. As expected, PAH crippled intracellular DNA-induced IRF3-responsive gene expression in a dose-dependent manner in all three cell lines (**Figures 1C–E**). Consistently, PAH markedly decreased the induction of the same set of genes triggered by HSV-1 in these cell lines (**Figure 1F**). The inhibitory activity of PAH on DNA-induced type I IFN production was not the result of cytotoxicity, as PAH had no effect on the cell viability at the used dosage. Collectively, these data suggest that PAH is a specific inhibitor of the DNA-sensing signaling pathway.

### PAH Directly Inhibits cGAS Activity

**Figure 2:**
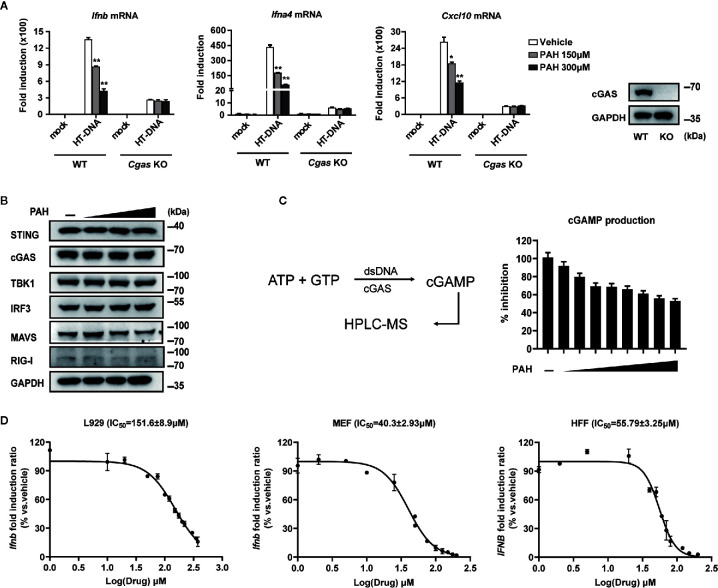
PAH directly inhibits cGAS activity. **(A)** WT or *Cgas*-deficient L929 cells were treated with Vehicle or PAH for 6 h and then stimulated with HT-DNA (4 μg•ml^−1^) for 6 h. The induction of *Ifnb*, *Ifna4*, and *Cxcl10* mRNA expression was then measured by qPCR (left panel). The cGAS protein levels were examined by immunoblot (right panel). **(B)** L929 cells were treated with a Vehicle or different PAH doses (0 to 300 μM) for 6 h, and then the cell lysates were immunoblotted with the indicated antibodies. **(C)** Schematic of dsDNA-dependent cGAS synthesis of cGAMP and measurement by HPLC-MS (left panel). The cGAMP production was measured in the presence of a Vehicle or different PAH doses (0 to 40 μM) (right panel). **(D)** L929, MEF, and HFF cells were treated with indicated concentrations of PAH for 6 h, followed by stimulation with HT-DNA for 6 h. The induction of *Ifnb* mRNA expression was then measured by qPCR. All of the experiments were repeated at least three times. Data in **(A, C, D)** are presented as mean ± SD. *P < 0.05, **P < 0.01.

As cGAS is the primary intracellular DNA sensor, we suspected that PAH inhibited the DNA-sensing pathway by targeting cGAS. We obtained *Cgas*-deficient L929 cells *via* CRISPR-Cas9-mediated targeting to test whether PAH suppressed cGAS-mediated DNA-sensing signaling. Administration of PAH impaired the DNA-induced antiviral gene expression in WT L929, whereas such inhibitory effect vanished in *Cgas*-deficient L929 cells (**Figure 2A**), indicating the inhibition of the DNA-sensing pathway by PAH required the presence of cGAS.

Next, we tested whether PAH regulated the protein levels of cGAS and other essential components for the host’s innate defense against viruses. As illustrated in **Figure 2B**, PAH did not affect the endogenous protein levels of cGAS and other molecules involved in cGAS-STING/RIG-MAVS signaling, suggesting the inhibitory effect of PAH on the DNA-sensing pathway to be a consequence of impaired cGAS enzyme activity. We performed *in vitro* dose-titration experiments with recombinant human cGAS and measured cGAMP production using HPLC-MS to test this hypothesis (**Figure 2C**). As expected, PAH effectively inhibited cGAMP production with a biochemical IC_50_ (half maximal inhibitory concentration) value of 31.3 μM (**Figure 2C**). To further determine the physiological inhibition of PAH on cGAS-mediated signaling, we performed concentration–response analysis in different mouse and human cell lines. As determined by RT-qPCR, PAH showed dose-dependent inhibition on the *Ifnb*/*IFNB* expression with cellular IC_50_ of 151.6 ± 8.9 μM for L929, 40.3 ± 2.93 μM for MEF, and 55.79 ± 3.25 μM for HFF cells (**Figure 2D**). Altogether, these data indicate that PAH suppresses DNA-induced type I IFN signaling by directly inhibiting the enzymatic activity of cGAS.

### PAH Suppresses cGAS-Mediated Innate Immune Response

**Figure 3:**
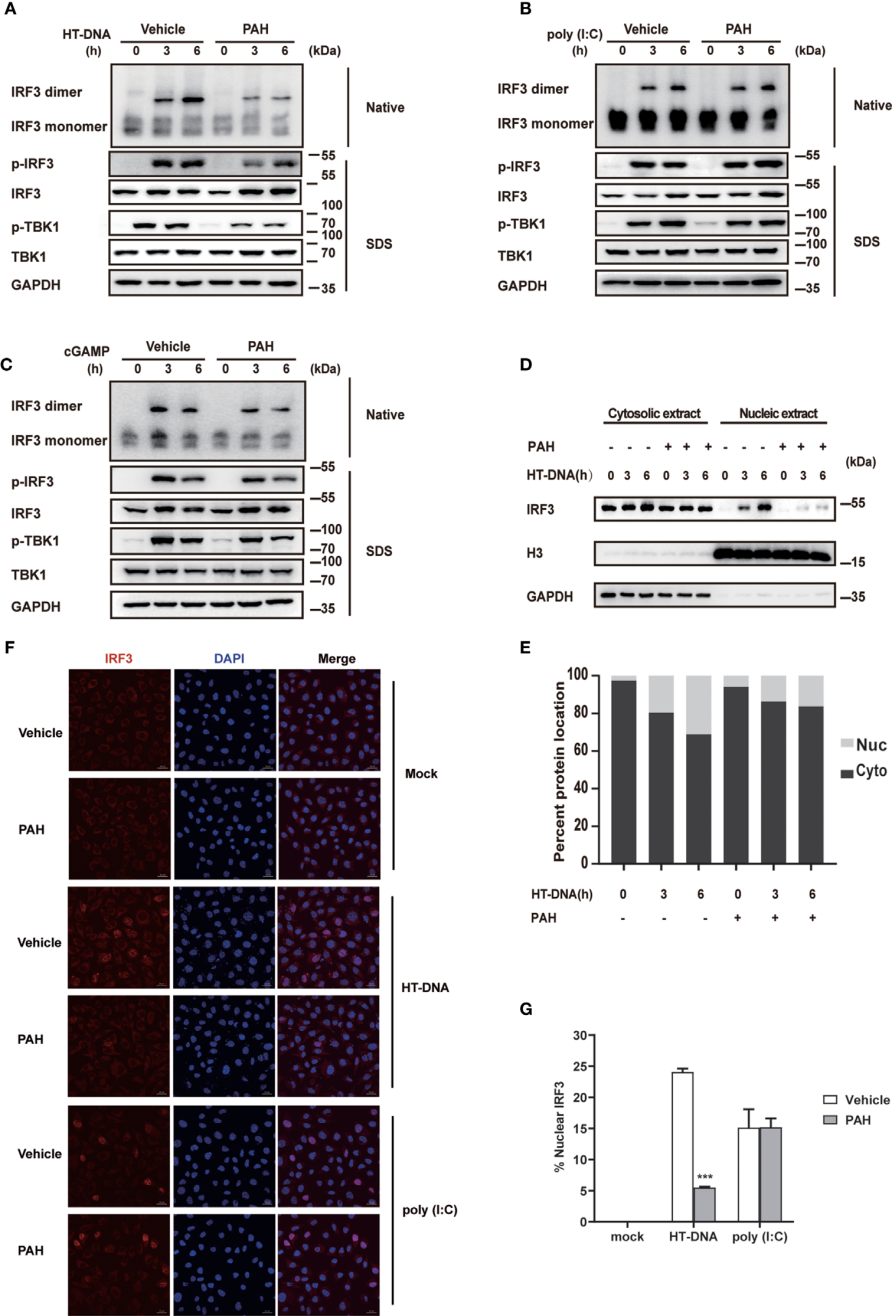
PAH suppresses cGAS-mediated innate immune response. **(A–C)** L929 cells were treated with Vehicle or PAH (200 μM) for 6 h and then stimulated with HT-DNA (4 μg•ml^−1^) **(A)**, poly(I:C) (3 μg•ml^−1^) **(B)**, or cGAMP (1 μg•ml^−1^) **(C)** for the indicated times. The cell extracts were analyzed for TBK1 and IRF3 phosphorylation by SDS-PAGE or IRF3 dimerization by native PAGE. **(D, E)** L929 cells were treated with Vehicle or PAH (200 μM) for 6 h and then stimulated with HT-DNA (4 μg•ml^−1^) for the indicated times. The IRF3 protein levels in cytoplasmic (Cyto) and nuclear (Nuc) fractions were analyzed by immunoblot **(D)**. The relative ratio of indicated proteins in Cyto and Nuc was analyzed by Image J **(E)**. **(F)** L929 cells were treated with Vehicle or PAH (200 μM) for 6 h, and then stimulated with HT-DNA (4 μg•ml^−1^) or poly(I:C) (3 μg•ml^−1^) for 4 h before staining with an antibody against IRF3 (red) and imaged by confocal microscopy. Scale bars represent 10 μm. **(G)** The relative ratio of nuclear IRF3 from **(F)** was analyzed by Image J. All of the experiments were repeated at least three times. Data in **(G)** are presented as mean ± SD. ***p < 0.001.

Upon sensing intracellular DNA, cGAS initiates a series of downstream signaling transduction events, including TBK1 phosphorylation and IRF3 phosphorylation/dimerization, critical for inducing the antiviral type I IFN responses. To explore whether PAH inhibited the activation of cGAS-mediated innate immune signaling, we checked the impact of PAH on these phenomena. Compared with the control group, PAH markedly attenuated the phosphorylation of TBK1 and IRF3 when stimulated with HT-DNA (**Figure 3A**). Likewise, native western blotting revealed that the dimerization of IRF3 was greatly impaired by PAH treatment (**Figure 3A**). In contrast, PAH had no or marginal effect on these processes when treated with poly(I:C) (**Figure 3B**) or cGAMP (**Figure 3C**), indicating PAH specifically suppressed DNA-induced downstream signaling transduction by targeting cGAS.

Next, we performed subcellular fractionation analysis to test whether PAH inhibited the nuclear translocation of IRF3. Consistent with the phosphorylation and dimerization results, PAH considerably decreased the nuclear translocation of IRF3. (**Figures 3D, E**). Confocal microscopy also revealed that PAH markedly suppressed HT-DNA-induced IRF3 nuclear translocation, whereas it did not affect that when stimulated with poly(I:C) (**Figures 3F, G**). Altogether, these data establish that PAH specifically inhibits the cGAS-mediated innate immune response.

### PAH Restricts the Host Innate Antiviral Defense *In Vitro*

**Figure 4:**
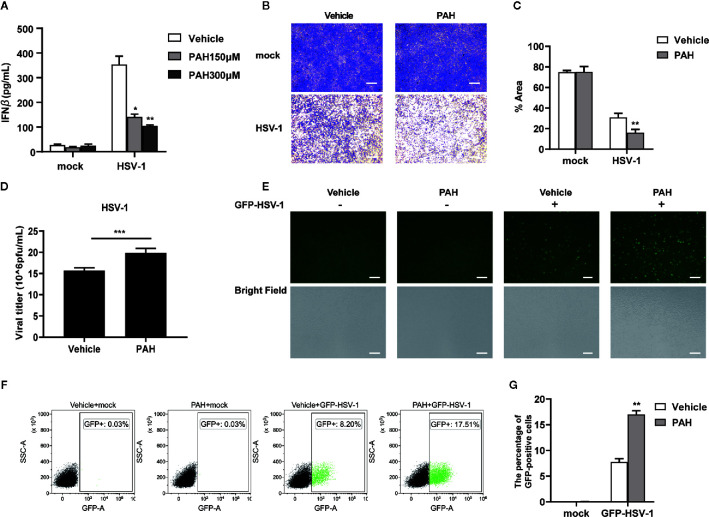
PAH restricts the host innate defense against HSV-1 *in vitro.* **(A)** L929 cells were treated with Vehicle or indicated PAH concentrations for 6 h and then stimulated with HSV-1 (MOI = 1) for 9 h. The supernatants were collected, and ELISA determined the amounts of IFN-*β*. **(B)** L929 cells were treated with Vehicle or PAH (200 μM) for 6 h and then infected with HSV-1 (MOI = 1). The proliferation of cells was examined by crystal violet staining. Scale bars represent 100 μm. **(C)** The statistical analysis of stained cell ratios from **(B)**. **(D)** L929 cells were treated with Vehicle or PAH (200 μM) for 6 h and then infected with HSV-1 (MOI = 1). The titers of HSV-1 were determined by standard plaque assay. **(E)** L929 cells were treated with Vehicle or PAH (200 μM) for 6 h and then infected with GFP-HSV-1 (MOI = 0.3) for 16 h. GFP-HSV-1 replication was visualized by fluorescence microscopy. Scale bars represent 100 μm. **(F)** L929 cells were treated with Vehicle or PAH (200 μM) for 6 h and then infected with GFP-HSV-1 (MOI = 0.3) for 16 h. GFP-HSV-1 replication was visualized by flow cytometry. **(G)** The statistical analysis of GFP-positive cell ratios from **(F)**. All of the experiments were repeated at least three times. Data in **(A, C, D, G)** are presented as mean ± SD. *P< 0.05, **P< 0.01, ***P< 0.001.

As IFN-*β* protects host cells against virus infection, we detected the secretion of IFN-*β* in cell supernatant *via* ELISA. Compared with the control group, PAH suppressed the IFN-*β* secretion in a dose-dependent manner upon stimulated with HT-DNA (**Figure S1**) or HSV-1 (**Figure 4A**). L929 cells were pre-incubated with PAH and then infected with HSV-1 to explore whether PAH impaired the host innate defense against HSV-1. As expected, L929 cells incubated with PAH were more susceptible to HSV-1 infection as determined by the crystal violet staining assay (**Figures 4B, C**). Consistently, PAH administration increased the titer of HSV-1 virus as quantified by the standard plaque assay (**Figure 4D**).

Moreover, we challenged L929 cells with GFP-HSV-1 virus to detect viral replication by fluorescence microscopy. Compared with the control group, treatment of PAH markedly impaired host defense against GFP-HSV-1 virus infection, as shown by the stronger GFP-positive signal and an increased number of the GFP-positive cells (**Figure 4E**). Consistently, FACS results also showed that treatment with PAH led to an increased number of GFP-positive cells (**Figures 4F, G**). Unexpectedly, PAH, to some extent, inhibited the host defense against VSV infection (**Figures S2A**–**F**), perhaps partially due to the protective role of cGAS in RNA virus infection (25, 26). These data reveal that PAH impairs the host innate defense against viruses by dampening IFN-I signaling *in vitro*.

### PAH Restricts the Host Innate Defense Against HSV-1 Infection *In Vivo*

**Figure 5:**
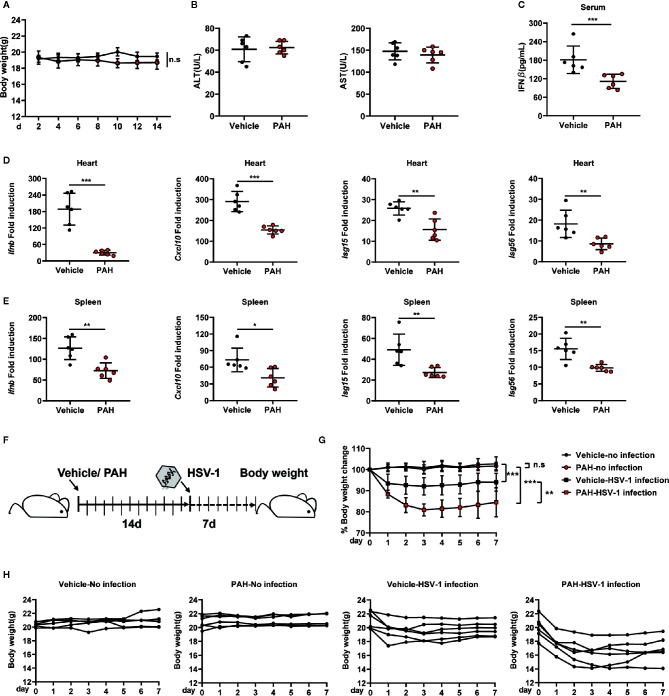
PAH restricts the host innate defense against HSV-1 infection *in vivo.* **(A)** Mice were orally administered Vehicle (0.03% CMC-Na solution) or 120 mg•kg^−1^ PAH once per day for 14 days, then injected with HSV-1 intravenously. The time course of mice body weight. **(B)** Mice were orally administered Vehicle (0.03% CMC-Na solution) or 120 mg•kg^−1^ PAH once per day for 14 days, then the levels of aspartate aminotransferase (AST) and alanine aminotransferase (ALT) in serum were measured at the 14^th^ day. **(C–E)** Mice were treated as in **(A)**. The concentrations of serum IFN-*β* of mice were measured by ELISA **(C)**, and the levels of *Ifnb*, *Cxcl10*, *Isg15*, and *Isg56* mRNA expression in the hearts **(D)** and spleens **(E)** of mice were measured by qPCR. **(F)** Schematic of drug administration and durable study after HSV-1 infection *in vivo.* **(G)** The time course of merged body weight from mice treated in **(F)**. **(H)** The time course of body weight from each mouse treated in **(F)**. Data in **(A–E, G–H)** are representative of two independent experiments (mean ± SD). n.s, no significance. *P < 0.05, **P < 0.01, ***P < 0.001.

The 8–12 weeks old WT mice were pretreated with PAH according to the previous research to explore the potential role of PAH on host defense against viral infection *in vivo* (15). Briefly, mice were orally administered with PAH or Vehicle (0.03% CMC-Na solution) at 120 mg•kg^−1^ body weight once per day for 2 weeks, followed by HSV-1 infection *via* tail vein injection (i.v.) for 12 h. Notably, PAH administration did not affect the mice’s body weight (**Figure 5A**). In addition, pretreatment with PAH for two weeks did not change the basal levels of serum aspartate aminotransferase (AST) as well as alanine aminotransferase (ALT) (**Figure 5B**), suggesting the safety and lack of *in vivo* toxicity for PAH under the used dosage. Next, we examined the production of IFN-*β in vivo*. Compared with the infected control mice, mice treated with PAH showed a more severe defect in serum IFN-*β* production after HSV-1 infection (**Figure 5C**). Consistently, the mRNA levels of *Ifnb* and ISGs (*Cxcl10*, *Isg15*, and *Isg56*) were severely crippled in the hearts and spleens of infected PAH-treated mice, as compared with the infected control mice (**Figures 5D, E**). It’s noteworthy that pretreatment with PAH did not affect the basal levels of serum IFN-*β* and the basal ISG mRNA levels of the hearts and spleens (**Figures S3A**–**C**), revealing that PAH did not change the *in vivo* immune circumstance.

Furthermore, we studied the durable anti-infection effect of PAH *in vivo*. Mice were pretreated with PAH as described above, followed by infection with HSV-1. Each mouse’s body weight was measured daily for another 7 days with drug administration (**Figure 5F**). There was no significant difference between PAH-treated mice’s body weight and that of control mice (**Figures 5G, H**), suggesting PAH itself had no *in vivo* toxicity. In comparison, infection with HSV-1 markedly decreased the body weight of Vehicle group mice. Moreover, treatment with PAH exacerbated the body weight loss after infection with the HSV-1 virus (**Figures 5G, H**). Altogether, these data suggest that PAH suppresses the host defense against HSV-1 infection *in vivo*.

### PAH Inhibits cGAS-Mediated Autoimmunity in Mice

**Figure 6:**
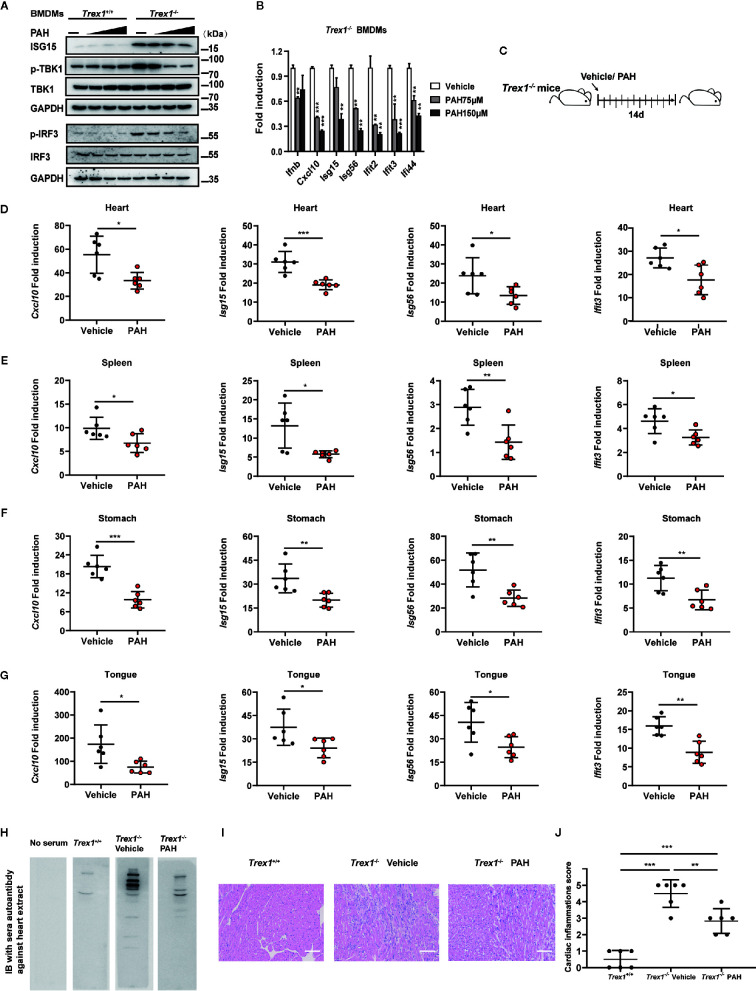
PAH inhibits cGAS-mediated autoimmunity in mice. **(A)** BMDMs from *Trex1* ^+/+^ or *Trex1^−/−^* mice were treated with different PAH doses (0 to 250 μM) for 6 h, then the cell lysates were immunoblotted with the indicated antibodies. **(B)** BMDMs from *Trex1^−/−^* mice were treated with indicated concentrations of PAH. The baseline of *Ifnb*, *Cxcl10*, *Isg15*, *Isg56*, *Ifit2*, *Ifit3*, and *Ifi44* mRNA expression was then measured by qPCR. **(C)** Schematic of *Trex1^−/−^* mice drug administration *in vivo.* 4 weeks old *Trex1^−/−^* mice were orally administered Vehicle (0.03% CMC-Na solution) or 120 mg• kg^−1^ PAH once per day for 14 days. **(D–G)** The levels of *Cxcl10*, *Isg15*, *Isg56*, and *Ifit3* mRNA expression in the heart **(D)**, spleen **(E)**, stomach **(F)**, and tongue **(G)** of mice treated in **(C)** were measured by qPCR. **(H)** Representative autoantibodies blotting band of heart extracts from WT mice. Heart extracts were blotted with serum from *Trex1* ^+/+^, *Trex1^−^* ^/^ *^−^* (Vehicle), and *Trex1^−^* ^/^ *^−^* (PAH) mice and detected using HRP-conjugated anti-mouse IgG. **(I)** Representative H&E-stained heart tissue sections from *Trex1* ^+/+^, *Trex1^–/–^* (Vehicle), and *Trex1^–/–^* (PAH) mice. Scale bars represent 100 μm. **(J)** Pathological scores of cardiac inflammations in *Trex1^−^* ^/^ *^−^* mice administrated with the Vehicle or PAH for 14 days. Data in **(A, B)** were repeated at least three times, data in **(D–J)** are representative of two independent experiments. Data in **(B, D–G)** are presented as mean ± SD. *P< 0.05, **P< 0.01, ***P < 0.001.

Abnormal activation of cGAS by self-DNA is linked to severe autoimmune diseases such as AGS, and inhibition of cGAS provides a powerful therapeutic strategy. As *Trex1^−/−^* mice are a tractable model to study AGS disease, we investigated whether PAH could be utilized to treat the autoimmune phenotype in *Trex1^−/−^* mice. First, we incubated BMDMs with different doses of PAH. Compared with BMDMs from WT mice, *Trex1^−/−^* BMDMs exhibited an aberrant upregulation of the ISG15 protein levels and the phosphorylation levels of IRF3 and TBK1 (**Figure 6A**). Administration of PAH decreased the levels of ISG15 protein and that of IRF3/TBK1 phosphorylation in a dose-dependent manner in *Trex1^−/−^* BMDMs (**Figure 6A**). Likewise, PAH effectively decreased self-DNA-induced expression of *Ifnb* and a panel of ISGs gene (*Cxcl10*, *Isg15*, *Isg56*, *Ifit2*, *Ifit3*, and *Ifi44*) in *Trex1^−/−^* BMDMs (**Figure 6B**).

To explore the potential therapeutic effect of PAH in cGAS-mediated autoimmune disease *in vivo*, 4 weeks old *Trex1^−/−^* mice were started to drug administration for up to 2 weeks as previously described (**Figure 6C**). Two weeks of treatment with PAH had no effect on the bodyweight of *Trex1^−/−^* mice (**Figures S4A**, **B**), revealing the *in vivo* safety of PAH in *Trex1^−/−^* mice. Then we examined the effects of PAH on the expression of ISGs in different tissues. As expected, PAH inhibited the expression of multiple ISGs genes (*Cxcl10*, *Isg15*, *Isg56*, and *Ifit3*) in the heart (**Figure 6D**), spleen (**Figure 6E**), stomach (**Figure 6F**), and tongue (**Figure 6G**) of *Trex1^−/−^* mice. Treatment with PAH decreased the abundance of sera autoantibodies (**Figure 6H**). Consistent with this observation, hematoxylin and eosin (H&E) and heart pathological scoring results showed that PAH administration could effectively alleviate the inflammation in the heart of *Trex1^−/−^* mice (**Figures 6I, J**). Collectively, these data reveal that inhibition of cGAS activity by PAH could potentially provide a therapeutic strategy for cGAS-mediated autoimmune diseases.

## Discussion

Recent researches have addressed the pivotal roles of cGAS in host innate defense against microbes such as bacteria (27), DNA viruses (28), RNA viruses (26) as well as retroviruses (29). Additionally, cGAS is the predominant sensor of mislocalized self-DNA from the nucleus or damaged mitochondrial (30), which resulted from many kinds of cellular or circumstance insults. The self-DNA sensing ability of cGAS has emerged as an important mechanism for developing inflammatory diseases and cancers. Thus small-molecular antagonists of cGAS may be useful for the treatment of diseases related to dysregulated cGAS signaling. In the present study, we identified that a natural monoterpenoid small molecule PAH could potently and selectively inhibit dsDNA-dependent cGAS enzymatic activity *in vitro* and *in vivo*. Our findings revealed that PAH might be beneficial in the therapy of cGAS-related autoimmune diseases.

Given its critical role in dsDNA-induced innate immunity, efforts to discover inhibitors of cGAS have been taken for the last few years, and several small molecules have been reported. By high-throughput screen with recombinant m-cGAS, RU.521 was identified as a specific inhibitor of cGAS enzyme without interfering with another innate immune signaling in both biochemical and cellular assays (31). Albeit its promise in mouse studies, RU.521 was demonstrated to be a poor inhibitor of human cGAS, underscoring the importance of species difference when screening cGAS inhibitors (32). In the later effort to discover human-cGAS-specific small-molecule inhibitors, G140 and G150 were identified to be potent and specific inhibitors of h-cGAS with cell-based activity (32). However, whether these compounds worked *in vivo* or had utility for the treatment of cGAS-related diseases were unknown. Combining high throughput cGAS fluorescence polarization (FP)-based assay and structure-based chemical optimization, PF-06928215, and related compounds were screened to inhibit cGAS enzymatic activity with high binding affinity as well as low IC_50_ values in the biochemical assay (33). Nevertheless, these compounds failed to inhibit cGAS activity in cellular assays for unknown reasons. Antimalarial drugs (AMDs) such as hydroxychloroquine (HCQ) were shown to interact with the cGAS/dsDNA complex by *in silico* screening and inhibit IFN-*β* production *in vitro*, although with a poor selectivity (34). Curiously, HCQ did not affect or even increased the ISG expression in the tissues of *Trex1^−/−^* mice or peripheral blood cells (PBMCs) from SLE patients (35). So far, the most promising cGAS inhibitor came from synthesized AMD derivative X6, which could attenuate the autoimmune disease phenotype in *Trex1^−/−^* mice (35). Given these observations, it is still urgent to develop new cGAS inhibitors with good efficiency/specificity and a safety profile. Here we characterized that PAH specifically inhibited cGAS activity *via* biochemical and cell-based assays. The suppression effect of PAH on cGAS-mediated innate immune signaling held in both mouse and human cells. *In vivo*, PAH impaired HSV-1-triggered antiviral gene expression and attenuated auto-inflammatory responses in *Trex1^−/−^* mice.

Consistent with our research, PAH was useful in ameliorating inflammatory diseases such as intestinal inflammation (15) and inflammatory skin diseases (19). As a perspective natural bioactive product, PAH shows several potential superiorities in future drug development. First, PAH exhibits beneficial therapeutic effects in several mouse disease models, including depression-like behavior (36), colitis inflammation (15), cancer (20), and atherosclerosis (37). Administration of PAH showed no side effects and toxicity in these models, revealing the biological safety of PAH *in vivo*. Second, the pharmacological effect of PAH has been improved *via* an optimized drug delivery system. Meanwhile, the pharmacokinetic parameters and tissue distribution of PAH have been reported previously (38).

It’s noteworthy that there are some limitations of our study. First, treatment with PAH did not eliminate the pathological autoimmune phenotype in *Trex1^−/−^* mice, and little is known about whether PAH can prolong the survival of *Trex1^–/–^* mice. A longer period of treatment or treatment with mothers of pups immediately after birth would help understand the outcomes. Although the IC_50_ values of cGAS inhibitors are cell-type dependent, the cellular IC_50_ values of PAH seem to be high. One possible explanation is the poor water solubility or cell penetration. Chemical optimization or a proper solvent may be helpful for PAH to overcome the membrane barriers. Third, we do not yet understand the inhibitory mechanisms of PAH. As PAH showed an inhibitory effect on both mouse and human cGAS, we suspected that PAH structurally disrupted cGAS binding with dsDNA or functioned on the conserved active sites of cGAS. The exact mechanism(s) responsible remains to be determined. The co-crystal structural and interdisciplinary approach will be necessary to dissect how PAH inhibited the cGAS activity.

PAH can also be considered for the treatment of other cGAS-related diseases such as cancer. The cGAS-STING-mediated inflammatory response can promote tumorigenesis and metastasis in a tumor-type-dependent and stage-specific manner. For example, the carcinogen DMBA could induce nuclear DNA leakage and activate cGAS-STING-dependent cytokine production, supporting inflammation-driven skin carcinogenesis (39). cGAMP produced in brain metastatic breast and lung cancer cells could transfer to the astrocyte and activated the STING pathway, promoting tumor growth and chemo-resistance (40). In chromosomal instability tumor cells, genomic DNA existed in cytoplasm activated cGAS-STING and downstream NF-κB signaling, promoting tumor cell invasion and metastasis (41). Indeed, PAH showed an inhibitory effect on gastric cancer growth by inducing cell autophagy (20). Considering that the activation of cGAS-STING signaling is implicated in some types of cancer, it is perspective that PAH may have an application in treating cancers.

## Data Availability Statement

The raw data supporting the conclusions of this article will be made available by the authors without undue reservation.

## Ethics Statement

The animal study was reviewed and approved by the Institutional Animal Care and Use Committee of China Pharmaceutical University and the Institutional Ethics Committee of China Pharmaceutical University.

## Author Contributions

SC and CW conceived the experiments. LC, CheL, YL, QY, HY, ChuL, WM, JZ, QW and SC conducted the experiments. SC and LC analyzed the results. SC and CW wrote the manuscript. All authors contributed to the article and approved the submitted version.

## Funding

This study is supported by the National Natural Science Foundation of China (31801076, 81802000), the Natural Science Foundation of the Jiangsu province (BK20180555), the China Postdoctoral Science Foundation (2018M630641), the National Key R&D Program of China (2016YFA0501800), the Project Program of State Key Laboratory of Natural Medicines (SKLNMZZ202002), the “Double First-Class” Project of China Pharmaceutical University (CPU2018GF10).

## Conflict of Interest

The authors declare that the research was conducted in the absence of any commercial or financial relationships that could be construed as a potential conflict of interest.

## Supplementary Material

Supplementary Figure 1 PAH Suppresses HT-DNA-triggered IFN-*β* Secretion. L929 cells were treated with Vehicle or indicated PAH concentrations for 6 h, and then stimulated with HT-DNA (4 μg•ml^−1^) for 9 h. The supernatants were collected, and ELISA determined the amounts of IFN-*β*. Data are representative of three independent experiments (mean ± SD). ****P* < 0.001.

Supplementary Figure 2 PAH Restricts the Host Innate Defense against VSV *in vitro.* **(A)** L929 cells were treated with Vehicle or PAH for 6 h and then infected with VSV (MOI = 0.5) for 6 h. The induction of *Ifnb*, *Ifna4*, and *Cxcl10* mRNA expression was then measured by qPCR. **(B)** L929 cells were treated with Vehicle or PAH (200 μM) for 6 h and then infected with VSV (MOI = 1). The proliferation of cells was examined by crystal violet staining. Scale bars represent 100 μm. **(C)** The statistical analysis of stained cell ratios from **(B)**. **(D)** L929 cells were treated with Vehicle or PAH (200 μM) for 6 h and then infected with VSV (MOI = 1). The titers of VSV were determined by standard plaque assay. **(E)** L929 cells were treated with Vehicle or PAH (200 μM) for 6 h and then infected with GFP-VSV (MOI = 0.4) for 16 h. GFP-VSV replication was visualized by fluorescence microscopy. Scale bars represent 100 μm. **(F)** L929 cells were treated with Vehicle or PAH (200 μM) for 6 h and then infected with GFP-VSV (MOI = 0.4) for 16 h. GFP-VSV replication was visualized by flow cytometry. **(G)** The statistical analysis of GFP-positive cell ratios from (F). All of the experiments were repeated at least three times. Data in **(A, C, D, F)** are presented as mean ± SD. **P*< 0.05, ****P*< 0.001.

Supplementary Figure 3 The Effect of PAH on Basal Immune Circumstance *in vivo.* **(A)** Mice were orally administered with Vehicle (0.03% CMC-Na solution) or 120 mg• kg^−1^ PAH once per day for 14 days, then the concentrations of serum IFN-*β* of mice were measured by ELISA. **(B, C)** The levels of *Ifnb*, *Cxcl10*, *Isg15*, and *Isg56* mRNA expression in the hearts **(B)** and spleens **(C)** of mice from **(A)** were measured by qPCR. Data in **(A–C)** are representative of two independent experiments.

Supplementary Figure 4 The Time Course of Body Weight from *Trex1^−/−^* mice. **(A)** The time course of body weight from each *Trex1^−/−^* mouse treated in (**Figure 6C**). **(B)** The time course of merged body weight from mice treated in (**Figure 6C**). Data in **(A, B)** are representative of two independent experiments. Data in **(B)** are presented as mean ± SD.
