# Supplementary figures and images for: Perillaldehyde Inhibition of cGAS Reduces dsDNA-Induced Interferon Response
Source: Front Immunol. 2021 Apr 22;12:655637. doi: 10.3389/fimmu.2021.655637 (PMC8100446; doi:10.3389/fimmu.2021.655637)

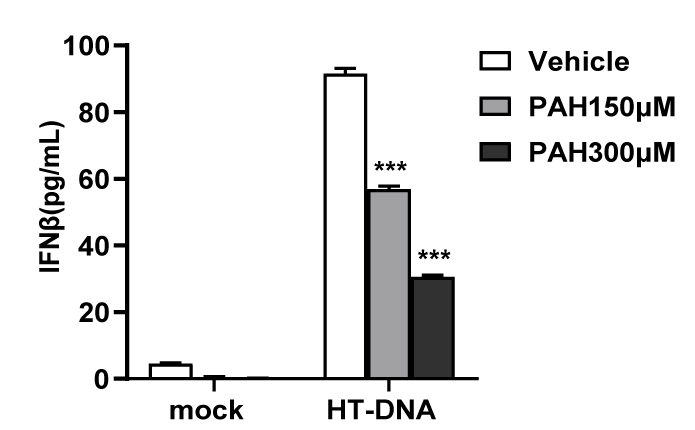

Supplement: Supplementary Figure 1 — PAH Suppresses HT-DNA-triggered IFN-β Secretion. L929 cells were treated with Vehicle or indicated PAH concentrations for 6 h, and then stimulated with HT-DNA (4 μg•ml−1) for 9 h. The supernatants were collected, and ELISA determined the amounts of IFN-β. Data are representative of three independent experiments (mean ± SD). ***P < 0.001. [file Image_1.tif]

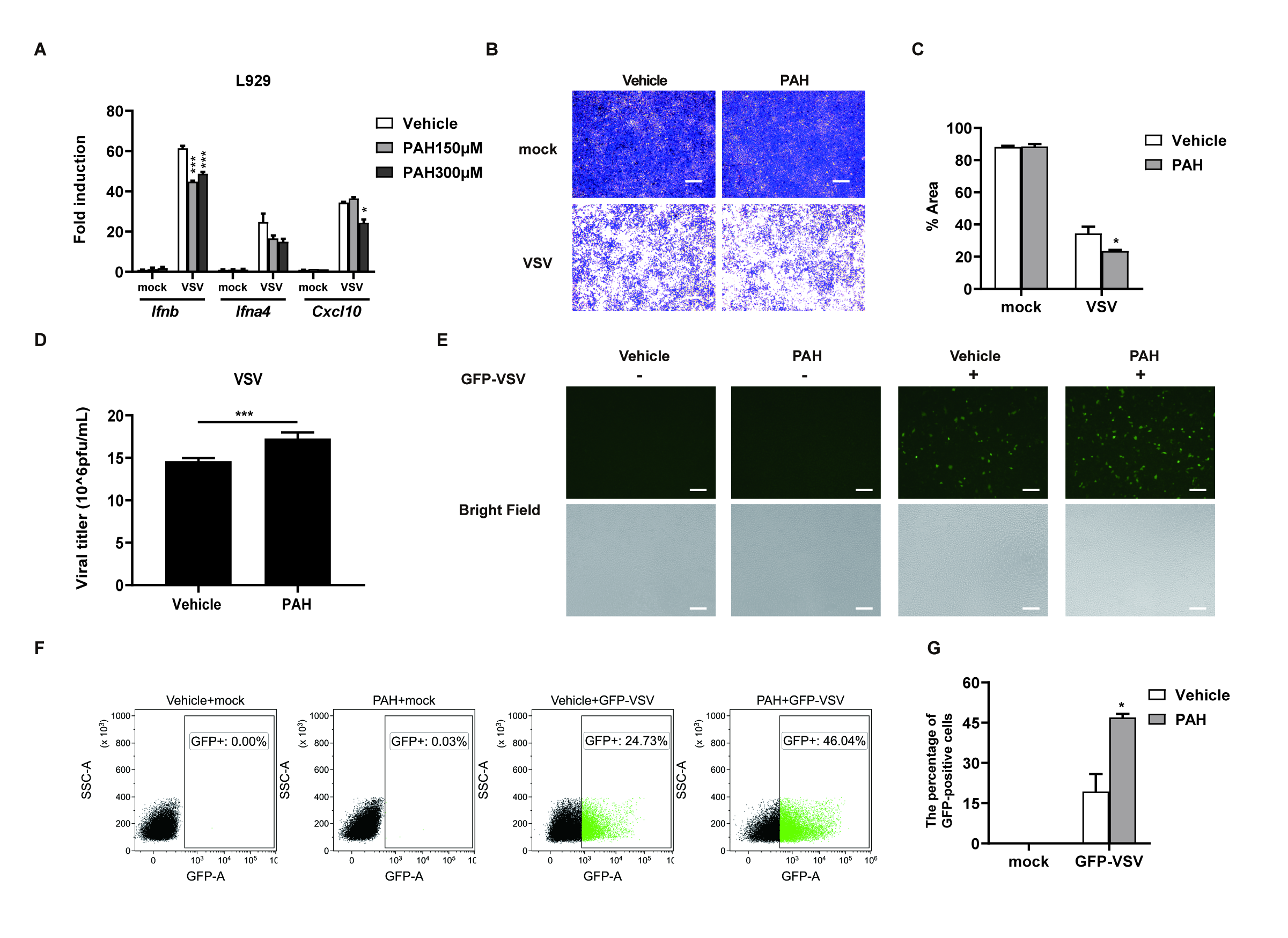

Supplement: Supplementary Figure 2 — PAH Restricts the Host Innate Defense against VSV in vitro. (A) L929 cells were treated with Vehicle or PAH for 6 h and then infected with VSV (MOI = 0.5) for 6 h. The induction of Ifnb, Ifna4, and Cxcl10 mRNA expression was then measured by qPCR. (B) L929 cells were treated with Vehicle or PAH (200 μM) for 6 h and then infected with VSV (MOI = 1). The proliferation of cells was examined by crystal violet staining. Scale bars represent 100 μm. (C) The statistical analysis of stained cell ratios from (B). (D) L929 cells were treated with Vehicle or PAH (200 μM) for 6 h and then infected with VSV (MOI = 1). The titers of VSV were determined by standard plaque assay. (E) L929 cells were treated with Vehicle or PAH (200 μM) for 6 h and then infected with GFP-VSV (MOI = 0.4) for 16 h. GFP-VSV replication was visualized by fluorescence microscopy. Scale bars represent 100 μm. (F) L929 cells were treated with Vehicle or PAH (200 μM) for 6 h and then infected with GFP-VSV (MOI = 0.4) for 16 h. GFP-VSV replication was visualized by flow cytometry. (G) The statistical analysis of GFP-positive cell ratios from (F). All of the experiments were repeated at least three times. Data in (A, C, D, F) are presented as mean ± SD. *P< 0.05, ***P< 0.001. [file Image_2.tif]

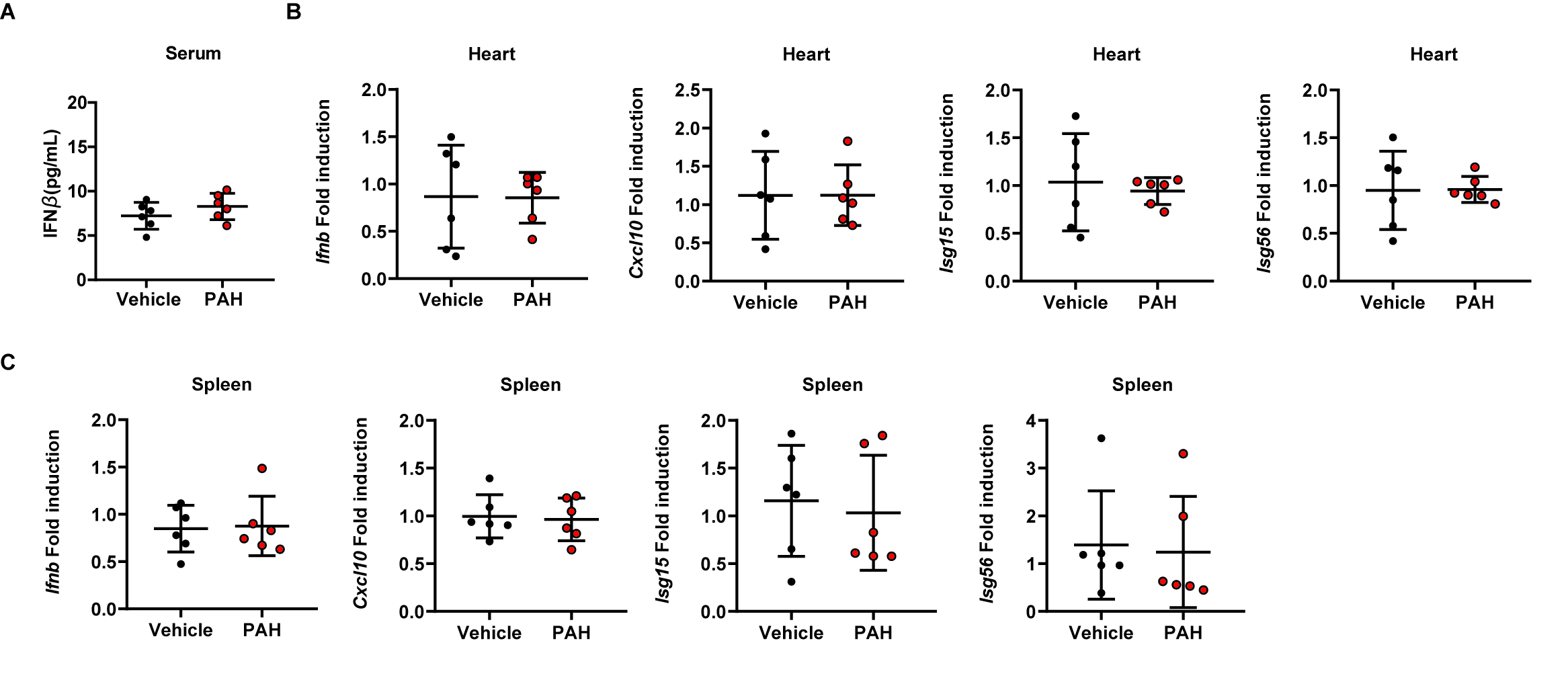

Supplement: Supplementary Figure 3 — The Effect of PAH on Basal Immune Circumstance in vivo. (A) Mice were orally administered with Vehicle (0.03% CMC-Na solution) or 120 mg• kg−1 PAH once per day for 14 days, then the concentrations of serum IFN-β of mice were measured by ELISA. (B, C) The levels of Ifnb, Cxcl10, Isg15, and Isg56 mRNA expression in the hearts (B) and spleens (C) of mice from (A) were measured by qPCR. Data in (A–C) are representative of two independent experiments. [file Image_3.tif]

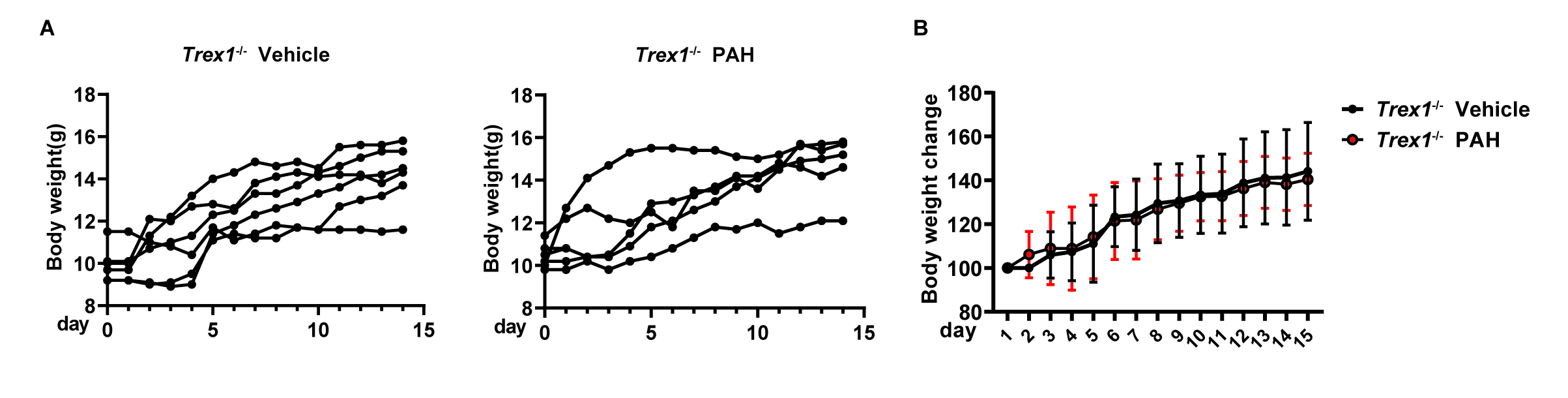

Supplement: Supplementary Figure 4 — The Time Course of Body Weight from Trex1−/− mice. (A) The time course of body weight from each Trex1−/− mouse treated in ( Figure 6C ). (B) The time course of merged body weight from mice treated in ( Figure 6C ). Data in (A, B) are representative of two independent experiments. Data in (B) are presented as mean ± SD. [file Image_4.tif]
